# Reliability of a Trunk Flexion and Extensor Muscle Strength Test with Hand-Held and Isokinetic Dynamometers in Female Athletes

**DOI:** 10.5114/jhk/172640

**Published:** 2023-11-28

**Authors:** Casto Juan-Recio, Amaya Prat-Luri, David Barbado, Francisco J. Vera-Garcia, Víctor Moreno-Pérez

**Affiliations:** 1 Sports Research Centre, Department of Sports Sciences, Miguel Hernández University of Elche, Alicante, Spain.

**Keywords:** field tests, isometric, core, force

## Abstract

An accurate trunk muscle strength assessment seems very important to design and individualize training and rehabilitation programs in clinical and sport settings. Hand-held dynamometers (HHDs) are interesting alternatives to isokinetic dynamometers for assessing trunk isometric muscle strength because they are inexpensive instruments and easy to use. This cross-sectional observational study aimed to examine the reliability of two novel sitting tests for assessing trunk flexion and extension isometric strength using an HHD and their relationship with two other novel isometric tests that use an isokinetic dynamometer. Twenty-four female amateur athletes (age: 24.5 ± 2.64 years; body height: 164.45 ± 6.33 cm; body mass: 63.17 ± 10.35 kg) participated in this study. A test-retest design was carried out one-week apart to examine the reliability. The relationship and the degree of agreement between the HHD and the isokinetic dynamometer measurements were analysed using Pearson correlation and Bland-Altman analysis, respectively. In general, the reliability of all isometric strength tests was good, with ICCs ranging from 0.65 to 0.87 and typical error < 15%. Pearson correlations were moderate, with values of r = 0.47 (R^2^ = 0.22) and r = 0.42 (R^2^ = 0.18) for flexion and extension strength, respectively. Bland-Altman plots showed no agreement between HHDs and isokinetic measurements. All trunk isometric tests using both, an isokinetic dynamometer and HHDs, provide reliable measurements for assessing trunk flexion and extension strength. According to the comparative analysis, both measurement types are different and cannot be used interchangeably. Health and sport professionals should choose the test that best suits the biomechanical characteristics required for functional goals or success in a given sport.

## Introduction

Reducing trunk muscle strength deficits and imbalances seems to be a relevant purpose to reduce the risk of low back pain and its severity (Cho et al., 2014; Mueller et al., 2012; Rossi et al., 2017; Steele et al., 2019). In addition, trunk muscle strength plays an important role in daily life activities (Estrázulas et al., 2020), preventing the elderly from suffering falls (Shahtahmassebi et al., 2017) and also contributing to athletic performance in many sports (Barbado et al., 2016; Correia et al., 2016; Evans et al., 2007). Thus, an accurate assessment of trunk muscle strength seems very important to design and individualize training and rehabilitation programs in clinical and sport settings.

Traditionally, isokinetic dynamometry is considered the gold standard for measuring isokinetic and isometric muscle strength (Estrázulas et al., 2020) through well standardized protocols that allow to control the type of contraction, angular velocity, range of motion, the angle of isometric contraction, etc. (Almutairi et al., 2023; Collado-Mateo et al., 2018; Gómez et al., 2005). In spite of its usefulness for assessing trunk muscle strength, isokinetic dynamometry protocols are time-consuming and they are also sophisticated procedures that require high-cost non-portable equipment and complex analyses (Harding et al., 2017), limiting their use in both clinical and field settings (Zouita Ben Moussa et al., 2020).

To overcome these drawbacks, portable devices like hand-held dynamometers (HHDs) are interesting alternatives for assessing trunk isometric muscle strength (Harding et al., 2017) because they are inexpensive instruments and easy to use. In this sense, previous research has already used some protocols with HHDs to assess trunk muscle strength (De Blaiser et al., 2018; Harding et al., 2017; Jubany et al., 2015; Moreno-Navarro et al., 2021). However, it seems there is not enough scientific evidence about which is the most valid and reliable protocol to measure isometric trunk muscle strength accurately as some limitations have been reported. For example, the position performed during the Biering-Sørensen test in which the examiner applies resistance over the interscapular region with the HHD is the most used test to assess isometric trunk extensor muscle strength. However, the standardization of this protocol is difficult (Demoulin et al., 2012) because of the variable degree of resistance on the HHDs provided by the examiner, which can affect the reliability of this test (0.24 < intraclass correlation coefficient (ICC) < 0.98) (De Blaiser et al., 2018; Moreno-Navarro et al., 2021; Vlažná et al., 2021). Due to poor reliability and the lack of standardization and functionality of these protocols that used a lying position against the gravity (Demoulin et al., 2012), the standing posture has been recently looked into (Harding et al., 2017; Jubany et al., 2015; Vlažná et al., 2021) to assess trunk extension strength, showing excellent levels of reliability (ICC > 0.90). However, the force generated by the trunk extensors in a standing position could be influenced by the involvement of the hip extensor muscles, mainly the hamstrings and gluteus muscles. Performing trunk strength tests in a sitting position might overcome some of the above-mentioned limitations as it reduces the hip flexor and extensor muscle contribution to the test performance (Morini et al., 2008), providing a more specific trunk flexion and extension muscle strength assessment. The sitting position has also been found to be more suitable to limit an overload of the lumbar spine (Morini et al., 2008). However, isometric trunk flexion strength tests in a sitting position have shown non-consistent reliability values (0.25 < ICC < 0.93) (De Blaiser et al., 2018; Moreland et al., 1997; Moreno-Navarro et al., 2021). These tests, in which the examiner applies resistance with the HHD close to the sternal notch region, seem to present similar standardization problems to those observed in trunk extension muscle strength protocols (Demoulin et al., 2012; Moreland et al., 1997). Therefore, new reliable and easy to standardize HHD protocols are needed for assessing isometric trunk flexion and extension strength in clinical and field settings.

Based on the above-mentioned limitations and the fact that most of the former studies using HHDs were performed on males, this study aimed: i) to examine the reliability of two novel sitting tests for assessing isometric trunk strength in healthy athletic females using an HHD with a well-standardized protocol that limits both, the lower limb contribution and the examiner’s influence on the measurement; ii) to examine the relationship between these novel isometric HHD tests and two novel tests for assessing isometric trunk strength using an isokinetic dynamometer.

## Methods

### Participants

Twenty-four amateur female athletes (age: 24.5 ± 2.64 years; body height: 164.45 ± 6.33 cm; body mass: 63.17 ± 10.35 kg) participated in this study. Participants performed 1–3 h of physical activity per day during 3–5 days per week, but they did not follow a structured trunk exercise program. Participants presenting urinary incontinence, inguinal hernia or a disease that contraindicated physical exercise practice (severe respiratory diseases, hypertension, heart disease, etc.) were excluded. Once the purpose and research procedure were explained to participants, they were asked to sign an informed consent form approved by the Miguel Hernández University of Elche Research Project Evaluator Office (approval code: DPS.FVG.01.18; approval date: 11 March 2019) according to the Declaration of Helsinki.

### Tests Description

Since there is no consensus on the best testing position, we examined different angles to those used by previous studies.

### Trunk Isometric Strength Tests Using a Hand-Held Dynamometer

**Figure 1:**
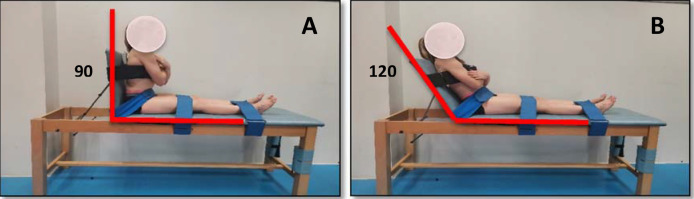
A participant performing trunk isometric strength tests using a hand-held dynamometer. A) Trunk extension; B) Trunk flexion

Two novel tests were used to evaluate the isometric trunk flexion and extension muscle strength using the Layfayette Manual Muscle Tester dynamometer (model 01165, Indiana, USA) (Figure 1). Trunk flexion strength was evaluated with participants seated on a stretcher with their knees extended to minimize the influence of the hip muscles, and supported on a mobile backrest that was placed at 120º with respect to the horizontal. The HHD was placed in the centre of the sternum using a non-extensible strap to reduce examiner’s participation. Regarding the trunk extension strength assessment, participants were placed in a similar sitting position, but with the mobile backrest placed on the vertical plane and the HHD on the centre of the scapulae also using a non-extensible strap. Participants were also cinched at the hip, thigh, and ankle height to reduce the involvement of the lower extremities in both tests. In each test, they performed five 5-s repetitions with a 10-s rest interval in between. The rest time between both tests was 5 min. The first two repetitions were performed progressively to familiarize participants with the test. Participants were then asked to perform their maximum isometric exertion in the last three repetitions. Participants were vigorously encouraged during each trial. HHD values were recorded in Newtons and the dynamometer activation threshold was set at 90 N. The strength values were converted into torque values (N•m) by considering the individual moment arm length (distance from the anterosuperior iliac crest to the centre of the HHD). The maximum torque (N•m) and the maximum strength (N) values obtained by participants in the second session in both tests were used for statistical analyses.

**Figure 2:**
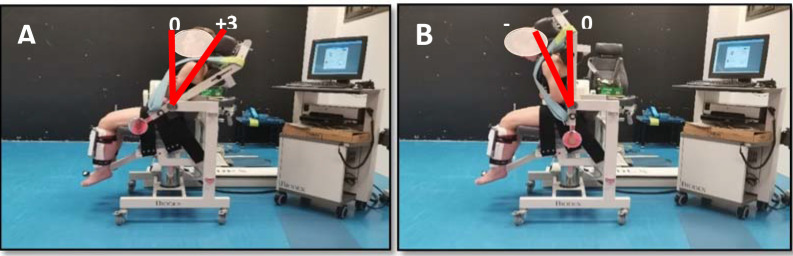
A participant performing trunk isometric strength tests using an isokinetic dynamometer.

Two novel tests were used to evaluate the maximum isometric strength of the trunk flexor and extensor muscles using the Biodex® isokinetic dynamometer (Model 2000, System 4 Pro; Biodex Corporation, Shirley, NY, USA). Participants were seated on the dynamometer with their knees flexed at 90º and the trunk, hips and legs cinched with non-extensible straps. For trunk flexion strength evaluation, the participant´s trunk was placed at an angle of +30º with respect to the vertical (0º), while for trunk extension strength evaluation it was placed with an angle of −20º with respect to the vertical (0º) (Figure 2). A previous pilot study exploring the use of several trunk angulations showed that these trunk angulations facilitated the trunk muscle force production. Both tests consisted of five 10-s repetitions with a 10-s rest interval in between and a 5-min rest interval between tests. As in the HHD tests, the first two repetitions were performed progressively so that participants became familiar with the test. In the last three repetitions, participants were asked to perform their maximum isometric exertion. Participants were vigorously encouraged during trials. The maximum torque obtained by participants in the second session in both tests was used for statistical analyses.

### Procedures

Participants performed the four trunk isometric strength tests in two 30-min testing sessions spaced 48 h apart. In the first session, participants performed the HHD tests, while the isokinetic dynamometer tests were carried out during the second session (Model 2000, System 4 Pro; Biodex Corporation, Shirley, NY, USA). In order to investigate the reliability of the measurements, a test-retest design was carried out repeating the two testing sessions one-week apart.

In each testing session, participants completed a warm-up before the strength tests which consisted of several core exercises: 5 pelvic circumductions in each direction, 10 pelvic retroversions-anteversions, 10 repetitions of the cat-camel exercise, 10 crunches, 10 extensions in a prone position on a stretcher and 15 s maintaining the front, right side, left side and back bridge positions.

### Statistical Analysis

Descriptive statistics (mean and standard deviation) were calculated for all variables. In addition, the ratios between flexion and extension strength values were calculated. The data normality was confirmed using the Kolmogorov-Smirnov test (*p* > 0.05). The ICC_(3.1)_ was estimated as an index of test-retest relative reliability (Weir, 2005), while the typical error and the minimum detectable change were calculated for absolute reliability analysis. The interpretation of the ICC was made based on the following values: excellent (0.90–1.00), good (0.70–0.89), moderate (0.50–0.69) and low (< 0.50) (Munro, 2005). The typical error was calculated as the standard deviation of the difference between session 1 and session 2 divided by $\sqrt{2}$, while the minimum detectable change was established as 1.5 times the typical error. The confidence intervals were set at 95% for both the ICC and the typical error. Student *t*-tests of paired samples were performed to evaluate the learning or repetition effects between testing sessions. Finally, two analyses were used to examine the relationship between the variables of the HHD tests and the isokinetic dynamometer tests. First, the Pearson correlation coefficients were calculated between the two methodologies, being categorised as follows: negligible (0.00–0.09), weak (0.10–0.39), moderate (0.40–0.69), strong (0.70–0.89) and very strong (0.90–1.00) (Overholser and Sowinski, 2008). Second, Bland-Altman plots with 95% limits of agreement (mean difference ± 1.96 standard deviation) were calculated to examine the degree of agreement between the HHD and the isokinetic dynamometer tests (Martin Bland and Altman, 1986). Then, one-sample *t*-tests were performed to analyse the systematic error or bias in the mean differences between the HHD tests and the isokinetic dynamometer tests. Finally, a linear regression analysis was performed to examine proportional bias and whether there was any trend for the differences between the two methodologies over the range of measurements. The program SPSS version 25.0 for Windows 7 (SPSS Inc., Chicago, IL, USA) was used for the statistical analysis and the significance level was set at *p* < 0.05.

## Results

The relative reliability for the isometric strength variables was mostly good with ICCs ranging from 0.65 to 0.87 (Table 1). Regarding the absolute reliability of the HHD tests, the typical error was 8.13 N•m and 26.04 N (13.09%) and 17.72 N•m and 56.89 N (14.65%) for trunk flexion and extension isometric strength, respectively. Besides, the typical error for trunk flexion and extension isometric strength evaluated with isokinetic dynamometer was 12.21 N•m (10.72%) and 15.77 N•m (9.04%), respectively. Paired samples *t*-tests showed significant differences between test and retest measurements in all the isometric strength variables (negative differences for trunk extension strength in both methods and positive for trunk flexion strength using HHDs) except for the trunk flexion strength measurements obtained with the isokinetic dynamometer.

**Table 1:** Descriptive statistics (mean ± SD) and relative and absolute reliability for the trunk isometric strength variables.

		Isometric strength variables		Mean ± SD	Change in mean (CL)	ICC_3.1_ (CL)	TE (CL)	%TE (%MDC)
**HAND-HELD DYNAMOMETER**	**MAXIMAL MOMENT OF FORCE**	Trunk Flexion (N•m)	Test	59.56 ± 13.99	5.17* (0.32–10.03)	0.73 (0.47–0.87)	8.13 (6.32–10.03)	13.09 (19.64)
			Retest	64.73 ± 16.22				
		Trunk Extension (N•m)	Test	127.76 ± 38.88	−12.67* (−23.51–−1.83)	0.81 (0.61–0.92)	17.72 (13.71–25.08)	14.65 (21.98)
			Retest	113.73 ± 40.23				
	**MAXIMAL STRENGTH**	Trunk Flexion (N)	Test	190.20 ± 40.41	15.88* (0.33–31.43)	0.65 (0.34–0.83)	26.04 (20.24–36.53)	13.09 (19.64)
			Retest	206.08 ± 45.42				
		Trunk Extension (N)	Test	408.12 ± 117.80	−39.14* (−73.94–−4.35)	0.80 (0.59–0.91)	56.89 (44.00–80.52)	14.65 (21.98)
			Retest	366.25 ± 129.13				
		Ratio Flexion/Extension	Test	0.51 ± 0.21	0.11* (0.01–0.20)	0.73 (0.45–0.87)	0.16 (0.12–0.22)	27.58 (41.37)
			Retest	0.65 ± 0.36				
**ISOKINETIC DYNAMOMETER**		Trunk Flexion (N•m)	Test	113.21 ± 28.01	0.61 (−5.89–7.11)	0.79 (0.60–89)	12.21 (9.74–16.57)	10.72 (16.08)
			Retest	114.30 ± 23.28				
		Trunk Extension (N•m)	Test	181.30 ± 42.2	−10.18* (−18.57–−1.78)	0.87 (0.74–0.93)	15.77 (9.74–16.57)	9.04 (13.56)
			Retest	167.30 ± 40.5				
		Ratio Flexion/Extension	Test	0.66 ± 0.23	0.04 (−0.03–0.11)	0.75 (0.49–0.89)	0.11 (0.08–0.15)	15.94 (23.91)
			Retest	0.71 ± 0.19				

SD: standard deviation; CL: confidence limits; ICC_(3.1)_: intraclass correlation coefficient; TE: typical error; MDC: minimum detectable change; * Significance *p <* 0.05

The mean flexion/extension strength ratio obtained with the manual dynamometer was 0.51 and 0.65 (first and second session, respectively) with an ICC of 0.73 and a typical error of 0.16 (27.58%) (Table 1). Regarding the flexion/extension strength ratio obtained with the isokinetic dynamometer, it was 0.66 in the first session and 0.71 in the second session with an ICC of 0.75 and a typical error of 0.11 (15.94%).

Pearson correlations between HHD measurements and those obtained with an isokinetic dynamometer were moderate with values of *r* = 0.47 (*p* < 0.05) for flexion tests and *r* = 0.42 (*p* < 0.05) for extension tests, indicating that despite both assess accurately trunk strength, the differences in the protocols may have influenced these results.

**Figure 3:**
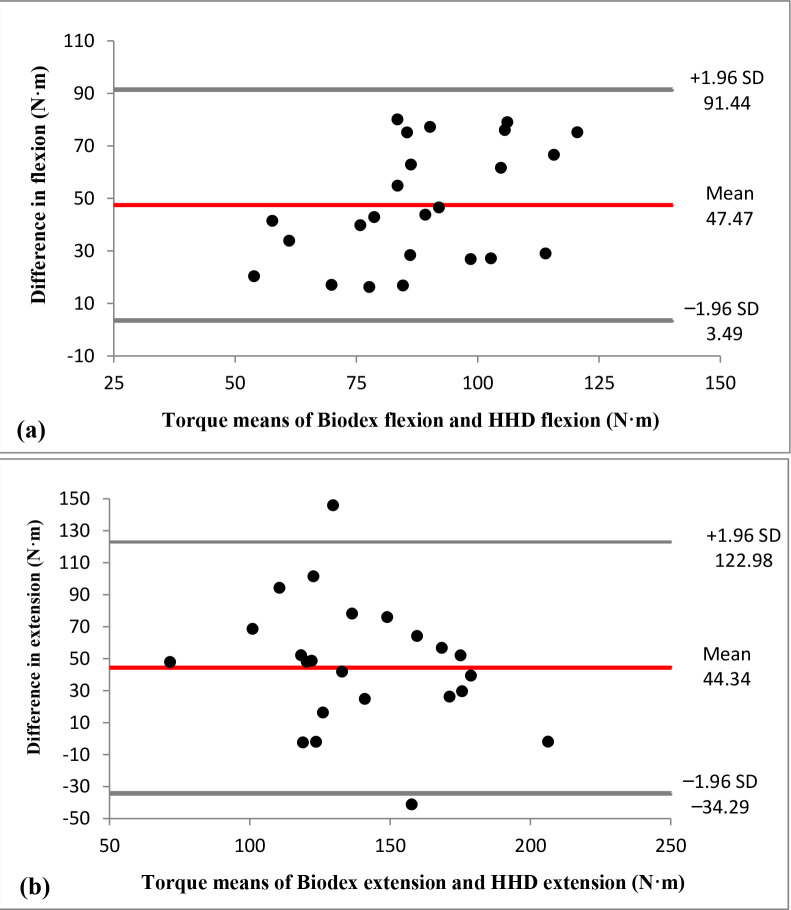
Bland-Altman plots for (a) trunk flexion strength tests º and (b) trunk extension strength tests in Biodex and a hand-held dynamometer. Mean difference between both methods and 95% limits of agreement are visualized with horizontal lines.

Bland-Altman plots are presented in Figure 3 (a and b). Regarding the trunk flexion strength tests, the one sample *t*-test showed a significant effect in the mean of differences between the HHD and the isokinetic dynamometer tests (*p* ≤ 0.001), as shown in the Bland-Altman plot (Figure 3a). This systematic bias of 47.47 N•m indicates significantly higher trunk flexion strength for isokinetic dynamometer measurements compared to the HHDs. In addition, linear regression between the two methods shows a slope of 0.57 (*p* = 0.027). This indicates proportional bias for this position and shows that the difference between the methods varies equally throughout the range of measurements. For these tests, the 95% limits of agreement varied between 3.49 N•m and 91.44 N•m.

Regarding the trunk extension strength tests, the mean difference of 44.34 N•m between the two methods (Figure 3b) was also significant (*p* ≤ 0.001), being again the isokinetic dynamometer measurements higher compared to the HHD measurements. In this case, the regression slope was not significantly different from zero (*p* = 0.192), indicating the absence of proportional bias, and the 95% limits of agreement ranged between −34.29 N•m and 122.98 N•m.

## Discussion

Due to the limitations of the isokinetic dynamometry (i.e., high cost, complex data processing and analysis, etc.), and some isometric trunk tests using HHDs (i.e., lack of standardization, poor reliability, etc.), the main aim of this study was to examine the reliability of two novel sitting isometric tests for assessing isometric flexion and extension trunk strength using HHDs. In addition, the relationship between these two tests and two other novel isometric trunk strength tests using an isokinetic dynamometer was analysed. In general, good reliability was found for all tests showing their accuracy for assessing isometric flexion and extension trunk strength. Nonetheless, although both methods were reliable assessing trunk strength, moderate correlations were found between tests using HHDs and the isokinetic dynamometer, indicating that both measurements, although related, are different and therefore cannot be used interchangeably.

A test-retest design with one-week between measurements was conducted to study the consistency of the four novel isometric trunk flexion and extension strength tests. Regarding the isokinetic dynamometer tests, they showed good reliability with an ICC_(3.1)_ > 0.79 and a typical error around 10%. These results are similar to those observed in a previous review analysing the reliability of isometric trunk strength assessments using isokinetic dynamometers (ICC > 0.81; 5.9% < SEM < 23.0%) (Estrázulas et al., 2020). Importantly, the reliability results of the HHD trunk strength tests found in this study (ICC_(3.1)_ > 0.71; TE ≈ 14%) are comparable to those obtained with the Biodex® isokinetic device, which reinforces the good standardization obtained in these tests for assessing trunk flexion and extension strength. In general, these results are similar to previous studies using HHDs (0.67 < ICC < 0.98) (De Blaiser et al., 2018; Karthikbabu and Chakrapani, 2017; Moreno-Navarro et al., 2021; Vlažná et al., 2021). Higher reliability in the current study was expected since the protocol using HHDs tries to minimize the examiner’s participation using straps to fix the HHDs to the participant’s torso (Figure 1). However, it must be noted that some participants reported some pain in the contact area with the HHDs during the maximal isometric efforts, mainly during trunk extensions, which could reduce the consistency of the measurements. Possibly, using a foam support on the contact area could help to reduce participants’ pain and consequently, improve the reliability of these HHD trunk strength protocols.

Although the isokinetic dynamometer and HHD protocols in this study were quite similar (e.g., sitting position, similar angle in trunk extension and flexion), the strength values obtained through isokinetic dynamometer tests were higher than those obtained with HHD tests. This could be related to the fact that in the isokinetic dynamometer protocol, the knees were flexed at 90º and the leg fixations of the Biodex® device allowed the legs to act as leverage, which resulted in higher trunk strength values. Small differences in the test characteristics (i.e., trunk angle, leg position, etc.) lead to different trunk strength values, which shows the difficulty of comparing participants assessed with different isometric trunk strength tests.

On the other hand, the flexion/extension ratios obtained in this study through both methods were very similar (0.71 ± 0.19 with the isokinetic dynamometer vs. 0.66 ± 0.36 with HHDs). According to Mueller et al. (2012), ratios of trunk flexion to extension in healthy untrained adults usually range between 0.7 and 0.9, but in athletes the ratio tends to be between 0.5 and 0.7, which is consistent with our data. In this sense, despite the novel HHD tests have shown to be reliable, it would be necessary to analyse their relationship with the risk of developing musculoskeletal disorders such as low back pain, as trunk flexion/extension strength imbalance is considered a risk factor for suffering low back pain and its severity (Cho et al., 2014; Mueller et al., 2012; Rossi et al., 2017). Thus, future studies obtaining normative data in large samples should explore and confirm if these ratios can be used in clinical settings.

In order to examine the relationship between both methods, the correlation and the level of agreement between the HHD tests and the isokinetic dynamometer tests were examined. Moderate correlations were found between the measurements of each HHD test and the corresponding isokinetic dynamometer test, indicating that both methods for measuring isometric trunk muscle strength are related, but with a low coefficient of determination (22% and 17% for trunk flexion and extension strength, respectively). However, a higher correlation coefficient was expected since both these methods supposedly measure trunk strength under a very similar condition. Bland-Altman plots (Martin Bland and Altman, 1986) showed a significant systematic bias between both testing methods, finding lower HHD strength measurements compared to the Biodex® measurements. Concretely, the isokinetic dynamometer measured an average of 47.47 N•m more than HHDs for trunk flexion strength and of 44.34 N•m more for trunk extension strength. Finally, although there are no studies (comparative data between both methods or normative data for HHDs) to establish what differences between both methods would be acceptable in a clinical context, certainly, a visual inspection of 95% limits of agreement in both Bland-Altman plots shows a large range between the limits which implies a poor agreement between both methods (Giavarina, 2015). The lack of agreement between both trunk strength protocols indicates that they cannot be used interchangeably.

Some limitations should be considered. The current study was carried out in a population of healthy recreational female athletes. Therefore, the results from this study cannot be generalized to the general population and it would be interesting to test other populations to analyse the robustness of the results obtained. In addition, the pain reported by some participants in the contact area of the torso with HHDs during the execution could affect the HHD test reliability and the poor correlation between both testing methods. Future studies should adapt the support surface of the HHDs to the participants body morphology for a better comfort during maximal trunk efforts. Furthermore, as previously mentioned, the moderate correlations observed between both protocols warn us from using them indistinctly. In this sense, as the new HHD protocols are reliable, low-cost, and easy-to-use, and it would be interesting to analyse their relationship with performance and risk factor variables.

## Conclusions

Based on the results of this study, both isokinetic dynamometer and HHD tests provide reliable measurements for assessing isometric trunk flexion and extension strength. As comparative analysis showed, both testing methods are different. Even small differences in the test characteristics lead to different trunk strength values, which should be considered by clinicians and coaches when choosing a reference test or a novel one. Health and sport professionals should choose the test procedures that best suit the biomechanical characteristics required for functional goals or success in a given sport (specificity criteria), while taking other important criteria into account, such as reliability, cost and availability. Overall, the HHDs proposed are tests that are interesting to use when aiming to assess trunk flexion and extension strength while minimizing the contribution of lower limbs through low-cost, reliable, and easy-to-use tests.
